# The metabolic impact of small intestinal nutrient sensing

**DOI:** 10.1038/s41467-021-21235-y

**Published:** 2021-02-10

**Authors:** Frank A. Duca, T. M. Zaved Waise, Willem T. Peppler, Tony K. T. Lam

**Affiliations:** 1 BIO5 Institute, University of Arizona, Tucson, AZ USA; 2 School of Animal and Comparative Biomedical Sciences, University of Arizona, Tucson, AZ USA; 3 Toronto General Hospital Research Institute, UHN, Toronto, Canada; 4 Department of Physiology, University of Toronto, Toronto, Canada; 5 Department of Medicine, University of Toronto, Toronto, Canada; 6 Banting and Best Diabetes Centre, University of Toronto, Toronto, Canada

**Keywords:** Diabetes, Obesity

## Abstract

The gastrointestinal tract maintains energy and glucose homeostasis, in part through nutrient-sensing and subsequent signaling to the brain and other tissues. In this review, we highlight the role of small intestinal nutrient-sensing in metabolic homeostasis, and link high-fat feeding, obesity, and diabetes with perturbations in these gut-brain signaling pathways. We identify how lipids, carbohydrates, and proteins, initiate gut peptide release from the enteroendocrine cells through small intestinal sensing pathways, and how these peptides regulate food intake, glucose tolerance, and hepatic glucose production. Lastly, we highlight how the gut microbiota impact small intestinal nutrient-sensing in normal physiology, and in disease, pharmacological and surgical settings. Emerging evidence indicates that the molecular mechanisms of small intestinal nutrient sensing in metabolic homeostasis have physiological and pathological impact as well as therapeutic potential in obesity and diabetes.

## Abstract (web summary)

The gastrointestinal tract participates in maintaining metabolic homeostasis in part through nutrient-sensing and subsequent gut-brain signalling. Here the authors review the role of small intestinal nutrient-sensing in regulation of energy intake and systemic glucose metabolism, and link high-fat diet, obesity and diabetes with perturbations in these pathways.

## Introduction

An increase in high-calorie intake and a sedentary lifestyle have resulted in continually increased rates of obesity, with ~2 billion adults affected by overweight or obesity^1^. Given that 80% of individuals with diabetes are also affected by excess weight or obesity, it is no surprise that diabetes rates are rising unabated, with almost 10% of the global population diagnosed with diabetes^2^. These dire statistics, along with their associated health care costs, underscore the critical need for safe and effective therapeutic interventions. However, in spite of significant advances in furthering our understanding of the pathophysiology of metabolic disease, costly and invasive bariatric surgery remains the best current treatment for obesity and diabetes^3,4^. Besides bariatric surgery, many pharmacological agents available for treating people with diabetes and obesity work by manipulating the gut-derived hormone glucagon-like peptide-1 (GLP-1), altogether highlighting the importance of the gastrointestinal (GI) tract in developing interventions for diabetes and obesity.

The GI tract was once thought to be primarily a site for nutrient absorption. It is now, however, well-established that the GI tract detects nutrients and triggers integrative and biological responses involving endocrine and neural components. The GI tract represents the first site of interaction between a meal and the host, informing the central nervous system (CNS) about the size and composition of a meal. The gut also contains the enteric nervous system that responds to a meal by adjusting GI physiology to maximize and optimize digestion and absorption^5^. These GI nutrient-sensing mechanisms, together with the effects of the postprandial rise in glucose, lipids, and amino acids, are vital for energy and glucose homeostasis via direct and indirect neural and endocrine mechanisms at various organs. While the glucose-sensing pathways in other tissues, such as the pancreas, liver, and brain also impact metabolic regulation, they have been reviewed elsewhere^6–8^. This review will focus on the preabsorptive role that intestinal nutrients have on metabolic homeostasis through nutrient-sensing mechanisms, with an emphasis on the regulation of food intake and systemic glucose regulation. Furthermore, we will highlight how obesity and diabetic settings alter these pathways, and how host–microbe crosstalk via diet-induced changes in the small intestinal microbiome impacts these pathways.

## GI nutrient-sensing physiology

**Fig. 1:**
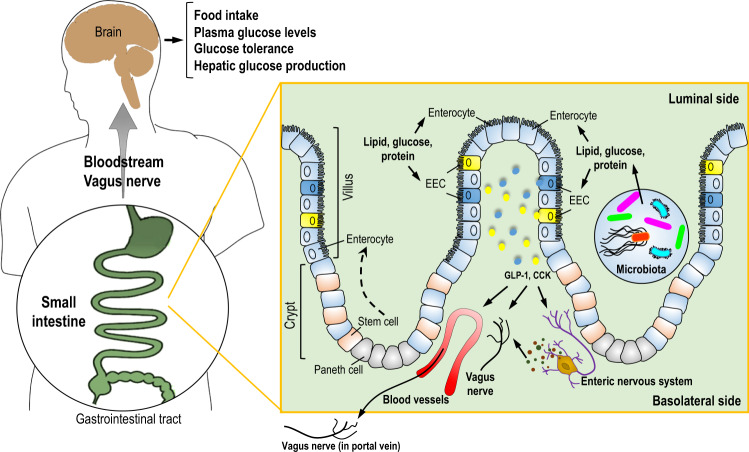
An integrated hormonal-dependent metabolic network that is activated by small intestinal nutrient sensing. Glucose, lipids, and protein from ingested foods are taken up by small intestinal enterocytes for cellular metabolism and absorption. Preabsorptive nutrients also activate enteroendocrine cells, triggering the release of GLP-1 and CCK. CCK and GLP-1 enter the circulation and act directly on peripheral organs to regulate metabolism. In parallel, CCK and GLP-1 act on the vagus nerve innervating the small intestine or portal vein as well as interact with the enteric nervous system to regulate glucose and energy homeostasis. In this context, the central nervous system can receive neuronal and/or direct hormonal signals to regulate feeding and maintain plasma glucose levels.

The GI tract consists of the small and large intestine, which differ in anatomy and function. The primary site of nutrient absorption takes place on the apical side of the polarized epithelial cell layer of the upper small intestine, while nutrients activate metabolic and sensory signaling pathways in the mucosal layer to exert whole-body biological responses before being absorbed into the circulation. Stem cells within the GI tract differentiate into multiple cell types, including secretory cells such as a hormone-producing and -secreting subtype enteroendocrine cells (EECs)^9^ (Fig. 1). EECs are scattered throughout the intestinal epithelium and are the key cell type that senses nutrients and initiates subsequent signaling by secretion of gut hormones. The classical rigid definition of EECs was based on the hormone they secrete in response to nutrient sensing, such as L-cells or I-cells secreting GLP-1 and cholecystokinin (CCK), respectively. However, EECs in reality exhibit a large degree of heterogeneity in hormone expression and secretion, as well as spatial expression along the GI tract as evident in single-cell RNA-Seq experiments^9–11^. Importantly, these studies examined small intestinal EECs^9–11^, highlighting the fact that a large population of EECs (i.e., cells secreting CCK and/or GLP-1) that is directly exposed to nutrients exists and enables nutrient sensing to exert whole-body biological responses via changes in gut peptides. Comparatively, there are high levels of EECs in the large intestine, but their activation may not be due to direct nutrient exposure, but rather from a neurohumoral reflex^12^ or other stimuli^13^.

Ingested nutrients trigger feedback mechanisms to prevent postprandial energy excess by suppressing food intake and endogenous nutrient production (Fig. 1) via the release of gut hormones. In addition to GLP-1 and CCK, EECs secrete up to 20 varieties of gut peptides that decrease energy intake and regulate glucose homeostasis. However, we will herein focus mostly on GLP-1 and CCK, the most studied small intestinal gut peptides that target the vagal afferents, brain, and pancreas to regulate energy and glucose homeostasis.

Although the post-prandial feedback mechanisms are partly coordinated by direct interaction of the liver, pancreas, and brain with circulating nutrients^6–8^, nutrient-induced small intestinal signaling mechanisms drive a majority of this feedback. For example, oral glucose administration with matching plasma glucose concentrations achieved by intravenous glucose administration causes up to three times more insulin to be released, and small intestinal nutrient-sensing pathways and the subsequent release of gut peptides mediate up to 80% of whole-body glucose disposal^14^. This is attributed to the glucoregulatory effect of GLP-1 and another gut peptide glucose-dependent insulinotropic polypeptide (GIP), in which the peptides are released from the small intestine in response to glucose sensing^15^. Recently, the role of the upper small intestinal GLP-1 secreting cells is highlighted by selectively knocking out *Gcg* expression (a gene from which GLP-1 is derived) in the lower gut (ileum and large intestine)^16^. In response to oral glucose challenge, distal *Gcg* knockout mice responded with normal levels of secreted plasma active GLP-1, thereby unveiling the importance of upper small intestinal GLP-1 secreting cells in glucose-sensing^16^.

### Nutrient sensing and signaling through the gut–brain axis

Small intestinal-derived peptides act in an endocrine fashion on the peripheral and CNS targets or in a paracrine fashion on vagal afferent neurons. These neurons are in close proximity to the gut, contain receptors for GLP-1, CCK, and peptide YY (PYY) and terminate in the nucleus of the solitary tract of the hindbrain where it has been demonstrated to regulate energy and glucose homeostasis^17^. Alternatively, gut peptides may activate the enteric nervous system to relay information to the vagal afferent terminals via glutamate or nitric oxide^18,19^, although the difficulty in targeting enteric neurons per se in vivo has limited the understanding of the role of the enteric nervous system in mediating the effects of nutrient-sensing. Nevertheless, the role of gut–brain vagal signaling pathways in metabolic homeostasis is demonstrated by the fact that chemical, surgical, genetic, or viral manipulation of the vagal gut–brain pathway impairs nutrient-induced regulation of energy and glucose homeostasis. This is reviewed in detail elsewhere^20^.

GLP-1R is expressed in the vagal afferent neurons, pancreas, and the brain^21^. Knockdown of the GLP-1R in vagal afferent neurons via lentiviral injection into the nodose ganglia (the inferior ganglion of the vagus nerve), which enables knockdown throughout the vagus nerve, increases meal size and postprandial glycemia, and blunts insulin release^22^ while knocking down GLP-1R via the use of transgenic mice in the gut–brain neuronal axis leads to increased glucose levels^23^. Similarly, knockdown of CCK-1R in the gut and/or the vagal afferent neurons disrupt glucose control and feeding in response to CCK administration^24,25^. In addition to the gut–brain metabolic axis governed by the glucose sensing in the small intestine, glucose sensing in the portal vein can be triggered by intestinal gluconeogenesis induced by protein and fiber intake and activate a portal vagal–brain axis to regulate glucose and energy homeostasis^26^. Overall, nutrient-sensing in the small intestine plays a major role in gut–brain negative feedback signaling that regulates energy and glucose homeostasis (Fig. 1), and in the following sections, we will highlight how sensing of specific nutrients regulates energy and glucose homeostasis to impact metabolic homeostasis.

### Small intestinal lipid sensing

#### Effect of lipid sensing on food intake

Small intestinal lipid sensing suppresses food intake through the release of CCK and GLP-1 and the activation of vagal afferents. It is traditionally believed that lipid sensing in the small intestine occurs on the apical luminal side where long-chain fatty acids (LCFA) are absorbed and metabolized to generate sensory anorectic signals. For example, intraduodenal infusion of Intralipid (a fat emulsion consisting mostly of linoleic acids) in rodents dose-dependently suppresses food intake within 10 min. The Intralipid effect is blocked by the local anesthetic tetracaine, which inhibits nerve impulses, indicating small intestinal vagal innervation as a mediator of the anorectic signals^27^. In support of a pre-absorptive lipid-sensing mechanism, intravenous infusion of Intralipid does not suppress food intake^28^, and the effects of duodenal Intralipid and/or linoleic acid occur independently of changes in circulating triglycerides^29,30^. Further, co-infusion of lipid with a lipase inhibitor blocks the ability of lipid to suppress food intake, as well as increase CCK and GLP-1^31^. However, the term “pre-absorptive lipid sensing” might be misleading as evidence suggests that the ability of intestinal lipid sensing to induce gut peptide release, activate vagal afferents, and reduce food intake is also dependent on chylomicron formation from LCFA and subsequent absorption to the basolateral side^32–34^. Inhibition of chylomicron formation with Pluronic L-81 attenuates the anorectic effect, celiac and cervical vagal afferent activation, and gut peptide release induced by lipids^33,34^. Similarly, infusion of fatty acids with a chain length of less than C10 (which do not assemble in chylomicrons but directly diffuse out of enterocytes) fails to reduce energy intake and increase CCK levels in humans^35^. These observations argue against the traditional view of lipid sensing that occurs on the apical luminal side of the small intestine, prior to absorption.

**Fig. 2:**
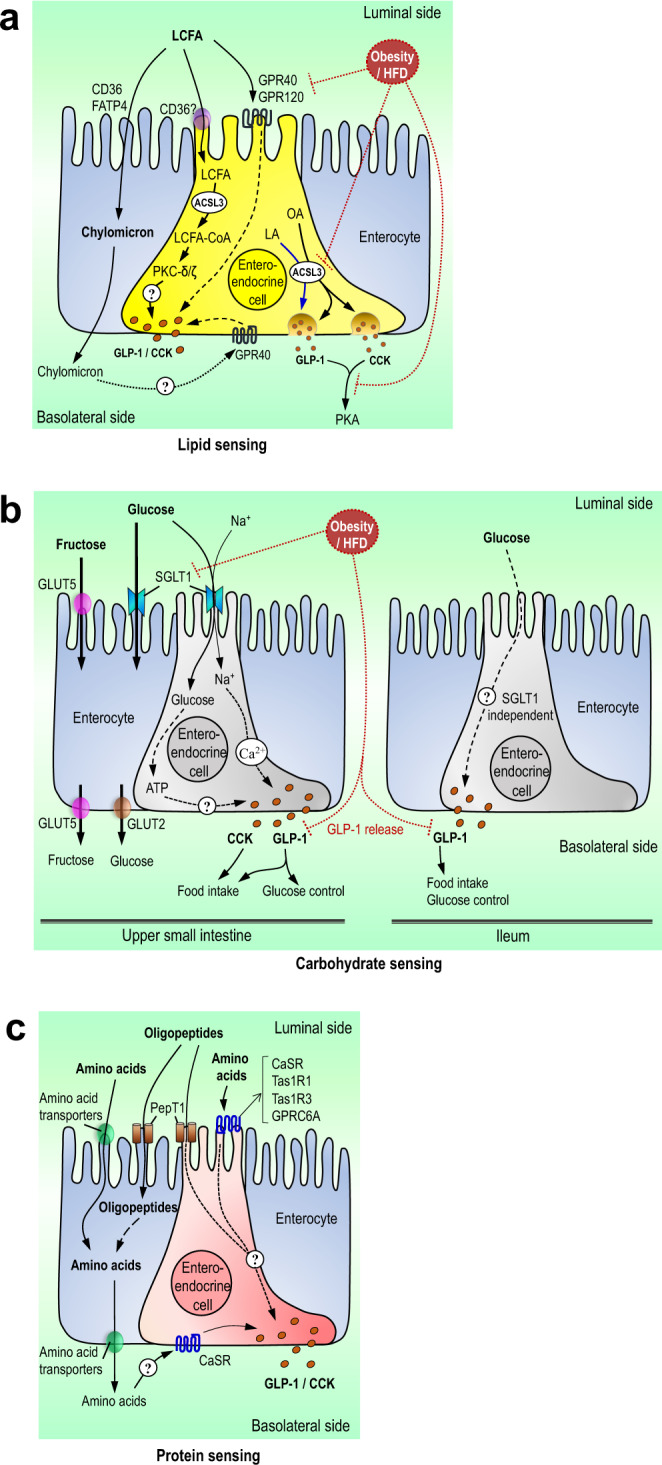
Mechanisms of small intestinal nutrient sensing. **a** Mechanisms of small intestinal lipid sensing. Small intestinal long-chain fatty acids are taken up (via CD36/FATP4 and/or simple diffusion) by enterocytes to form triglycerides and eventually packaged into chylomicrons released on the basolateral side. LCFA are also taken up by enteroendocrine cells to undergo ACSL3-dependent metabolism and activate PKCs to potentially stimulate CCK and/or GLP-1 release. Luminal LCFA may activate GPR40/120 to stimulate peptide release. The hydrolysis of chylomicrons by nearby enterocytes on the basolateral membrane may lead to increased LCFAs that can activate basolateral GPR40 to induce peptide release. In the setting of a high-fat diet or obesity, GPRs/ACSL3 expression, the release of GLP-1/CCK, and CCK signaling are reduced, leading to a disruption of fatty acid sensing. **b** Mechanisms of small intestinal carbohydrate sensing. Luminal glucose and fructose are transported into upper small intestinal enterocytes and/or enteroendocrine cells via SGLT1 and GLUT5, respectively. Through SGLT1, glucose directly and/or indirectly (via cellular metabolism) stimulates the release of gut peptides and regulates feeding and systemic glucose control in the upper small intestine. However, ileal glucose sensing may stimulate the release of GLP-1 independent of SGLT1. In response to high-fat feeding or obesity, small intestinal SGLT1 expression is reduced, leading to an impairment of glucose sensing, GLP-1 secretion, and glucose control. **c** Mechanisms of small intestinal protein sensing. Luminal small oligopeptides and amino acids are taken up by PepT1 and amino acid transporters, respectively, into the enterocyte and enteroendocrine cells. Small intestinal protein sensing stimulates the release of CCK and GLP-1 and regulates feeding and glucose homeostasis potentially via PepT1 dependent mechanisms. In addition, amino acids stimulate peptide release via the membrane-bound calcium-sensing receptor, the umami taste receptor, and G-protein-coupled receptor 6A. However, the downstream mechanism mediating the peptide release remains elusive. In parallel, amino acids are also transported to the basolateral side, and studies implicated that they may activate the calcium-sensing receptor to stimulate GLP-1 secretion.

Indeed, despite studies that elucidated the role of the receptor GPR40 (and GPR1120 to a lesser extent) in mediating LCFAs to stimulate gut peptides release^36^, the cellular site of action (apical vs. basolateral) has not been fully elucidated. Recently, utilizing an isolated intestinal perfusion model, it was shown that linoleic acid and GPR40 agonists induce GLP-1 release only when infused into the vasculature that would target the basolateral side, and not to the apical lumen^37^. In cultured, stable, immortalized murine EECs GLP-1 secretion is dependent on lipoprotein lipase to hydrolyze chylomicrons and on GPR40 to bind the liberated LCFAs^38^, further supporting that activation of EECs by LCFAs occurs on the basolateral side through hydrolysis of newly synthesized chylomicrons. Additionally, CD36 (a protein that transports fatty acids into cells and is documented to mediate chylomicron formation)^39^ knockout mice have impaired CCK release and fail to suppress food intake in response to duodenal lipids^40,41^, indicating that chylomicron formation in the small intestine is necessary for lipid sensing to lower food intake and thereby supporting the basolateral sensing hypothesis (Fig. 2a).

Vagal afferent fibers mediate the anorectic effects of intestinal lipid-sensing and are activated by several gut peptides^20^. Vagal afferents contain CCK-1 receptor (CCK-1R) and selective knockdown of CCK-1R in vagal afferents abolishes the ability of CCK to lower food intake^24^. However, the impact of vagal CCK-1R on small intestinal lipid-induced CCK signaling has not been established^24^. Vagal afferents also express GLP-1R^42^, and at least one study reports that GLP-1 signaling mediates the suppressive effects of jejunal linoleic acid infusion^43^. However, GLP-1R-expressing vagal afferent neurons were also reported to detect stomach and intestinal stretch but have no impact on nutrient-sensing^44^. Thus, the effect of GLP-1R on intestinal lipid sensing remains unclear. It is possible that the enteric nervous system, which contains GLP-1R, may mediate the gut–brain effect instead^18,19^.

#### Effect of lipid sensing on glucose homeostasis

Upper small intestinal infusion of Intralipid lowers plasma glucose levels and increases intravenous glucose tolerance within 50 min of infusion independent of a rise in plasma triglyceride, free fatty acids, and insulin levels^45,46^. Utilizing pancreatic–euglycemic clamps with plasma insulin levels maintained at a basal non-stimulated condition, upper small intestinal lipid infusion lowers hepatic glucose production^46^. These Intralipid effects are recapitulated in rats and mice that received specific LCFA infusion of either oleic (C18:1*n* − 9) or linoleic acid (C18:2*n* − 6)^45^. In contrast, a study with human participants reports that during the pancreatic–euglycemic clamps, no difference in glucose production is detected in response to intraduodenal infusion of lipid vs control group^47^. However, this observation is made in the presence of a progressive rise in plasma insulin and glucose levels prior to the start of the lipid or control infusion, and a parallel progressive drop in both plasma free fatty acids and glucose production in both groups^47^. Thus, it is not surprising that glucose production is not further lowered by intraduodenal lipid vs. saline infusion in a state that mimics the postprandial state, in which hepatic glucose production is already inhibited.

Similar to lipid-induced reductions in food intake, the ability of small intestinal lipid infusion to lower hepatic glucose production is dependent on CCK and GLP-1 signaling during the pancreatic (basal insulin)-euglycemic clamps^45,46^. Further, inhibiting CCK-1R signaling during refeeding, which activates nutrient-sensing pathways, results in postprandial hyperglycemia^25^. The specific mechanisms leading to the release of CCK and subsequent effects on glucose homeostasis are not fully understood, although the esterification of fatty acids to fatty acyl-CoA via acyl-CoA synthetase and the subsequent activation of mucosal protein kinase C (PKC)-δ are necessary for rats^46,48^. This is consistent with the fact that LCFA induces CCK release in intestinal secretin tumor cells via PKC-δ activation^49^. In parallel, the formation of chylomicron is also implicated in CCK release^50^, but the underlying mechanisms of how lipids stimulate CCK to release overall remain elusive (Fig. 2a). Further, the specific role of vagal GLP-1R signaling in mediating the glucoregulatory effect of lipids remains to be clarified.

In addition to lowering hepatic glucose production via a gut–brain axis^45,46^, small intestinal lipid-sensing could regulate glucose homeostasis via GLP-1-induced increase in insulin or suppression of glucagon secretion, as lipid-sensing increases GLP-1 release^45^^,^. However, GLP-1 induced increase in insulin secretion requires the presence of elevated circulating glucose levels^51^. Thus, it is possible that while an infusion of lipid alone increases GLP-1, this would not substantially elevate plasma insulin levels in the absence of a concomitant rise in blood glucose levels, as reported in human studies^52,53^. Despite this, increasing circulating active GLP-1 levels during an Intralipid intestinal infusion via DPP-IV inhibition (inhibits degradation of GLP-1) decreases glucose and increases insulin levels^52^. In addition, while GLP-1 is known to suppress glucagon secretion, glucagon is consistently increased with Intralipid infusion^52,53^. Although it has not been evaluated, this unexpected effect of Intralipid on glucagon could be due to the concurrent CCK release, as CCK lowers the inhibitory effect of glucose on glucagon secretion^54^.

#### Effect of metabolic dysregulation on intestinal lipid sensing

A high-fat diet (HFD) impairs lipid-induced gut–brain feedback regulating both energy and glucose homeostasis. Intestinal sensing of lipids is impaired during HFD in both rodents and humans^55^, however, it is still contentious as to whether this is due to chronic exposure to HFD or induction of obesity. For example, studies in rats have shown that the combination of an HFD with a genetic background that is predisposed to obesity, is associated with reduced intestinal-lipid sensing^56,57^. Furthermore, HFD decreases postprandial active GLP-1 and CCK levels in obese-prone rats compared to obese-resistant rats, potentially due to decreased intestinal expression of GPR40 and GPR120, receptors that are implicated in lipid-sensing induced secretion of gut peptides^56–58^ (Fig. 2a). The importance of interaction between diet and obesity for nutrient-sensing is also supported by human data. A 2-week high-fat dietary regimen in humans does not impair the suppressive effects on appetite or the CCK and GLP-1 response to an intralipid duodenal infusion^59^. However, individuals with obesity are less responsive to the satiating effects of dietary fat^60,61^. Obesity is also associated with reduced postprandial gut peptide levels^62^, and specifically for lipid-sensing, CCK release is blunted in individuals affected by obesity following intraduodenal oleic acid^63^. Therefore, future studies need to delineate the effect of diet vs. phenotype, which may be due to the ability of the gut microbiota to mediate this interaction between the diet and host physiology (discussed in more detail below).

Besides reductions in lipid-induced gut peptide release, it is possible that diminished sensitivity to gut peptides contributes to the reduced responsiveness to intestinal fat sensing in feeding control. The anorectic effect of CCK is impaired in HFD-fed mice and rats^55^, as is vagal afferent activation^55^, although this has not been fully examined in humans. Further, CCK-1R expression in vagal nerves is decreased in HFD induced obese rats^56^, ultimately contributing to reduced nodose ganglia cocaine and amphetamine-regulated transcript (CART) expression, a neuropeptide regulating energy homeostasis, in association with increased food intake and body weight^64^. However, vagal CCK resistance during obesity could also be due to obesity-associated leptin resistance, as the leptin receptor is co-expressed with CCK-1R in the vagal afferent neurons^65^ and leptin potentiates the suppressive effect of CCK on appetite and increases vagal afferent activation following CCK administration^66,67^. In addition, most studies demonstrate that the leptin-deficient, obese ob/ob mice and Zucker rats exhibit impaired responsiveness to CCK^68,69^. Using both genetic and viral approaches, the knockdown of leptin receptors in vagal afferent neurons impairs CCK responsiveness and induces hyperphagia^70^. Taken together, it is possible that impairments in CCK signaling both at the level of secretion and vagal activation could drive reduced lipid-induced satiation, although much of this remains to be tested in humans.

HFD also impairs the ability of upper small intestinal lipid sensing to improve glucose tolerance and lower hepatic glucose production^45,46^. The loss of effect of lipid-sensing following short-term 3-day HF feeding is partly due to impaired vagal CCK-1R signaling as both Intralipid and CCK (but not upstream activation of vagal protein kinase A) fail to lower glucose production in HF rats^25,46,71^. In parallel, HFD lowers upper small intestinal long-chain acyl-CoA synthetase-3 expression and disrupts long-chain acyl-CoA synthetase-3 dependent small intestinal fatty acid metabolism to regulate glucose homeostasis. However, transplantation of healthy microbiome to HF rats rescues the glucoregulatory effect of lipid-sensing via upregulation of long-chain acyl-CoA synthetase-3 expression in a small intestinal farnesoid x receptor (FXR) dependent fashion^45^. The underlying mechanism of how HF-induced changes in small intestinal microbiome alter bile acid pool, FXR, acyl-CoA synthetase-3, and lipid sensing remains elusive. Nonetheless, we hypothesize that enhancing long-chain acyl-CoA synthetase-3-dependent upper small intestinal fatty acid metabolism could increase GLP1 action to regulate glucose homeostasis in spite of CCK-1R vagal resistance (Fig. 2a).

### Small intestinal carbohydrate-sensing

#### Effect of carbohydrate-sensing on food intake

Intraduodenal infusion of glucose dose-dependently suppresses food intake in rodents^72^, and reduces food intake^73,74^ or favorably influences subjective appetite ratings^73^ in humans. Intravenous infusion of glucose to match the levels observed in blood following intestinal infusion of glucose does not inhibit food intake in rodents and humans^72,75^, highlighting the role of preabsorptive intestinal glucose-sensing. GLP-1R antagonist Exendin-9 abolishes the anorectic effect of both intragastric and voluntary sucrose loads in rats^76^, indicating that GLP-1 action mediates the effect of carbohydrate-sensing on food intake. Glucose-induced GLP-1 secretion from small intestinal EECs is dependent on sodium-glucose luminal transporter-1 (SGLT-1)^77^. SGLT-1 mediates the transport of glucose into the EECs, and the glucose transport is coupled with the transport of Na+ ions, causing membrane depolarization, entry of Ca2+ via voltage-gated calcium channels, and subsequent exocytosis of gut peptides on the basolateral membrane^78^. As non-metabolizable sugars transported via SGLT-1 also induce GLP-1 release^79^, glucose-sensing appears to be dependent on the transport of glucose via SGLT-1 but independent of subsequent cellular glucose metabolism. This finding has been confirmed in the human small intestine^80^. However, one cannot rule out that glucose metabolism and subsequent closure of ATP‐sensitive K^+^ channels could potentiate the response of EECs to glucose^81^ (Fig. 2b). In addition, a role for the sweet taste receptor (T1R2/T1R3), first observed in the oral cavity, was originally proposed to mediate sweet taste response in the small intestine as in vitro and in vivo studies reported the sweet taste receptor mediates GLP-1 release induced by glucose and non-caloric sweeteners^82^. However, recent studies report that non-caloric sweeteners do not induce GLP-1 release in primary L-cells and rodents^79,83^, and in humans, noncaloric sweeteners fail to induce gut peptide release and have no effect on appetite^84^.

It is possible that the suppressive effect of glucose on food intake depends on the specific site of the small intestine where glucose is sensed. For instance, a greater reduction of energy intake associates with higher CCK levels in humans receiving duodenal versus jejunal infusion of glucose^85^. However, in another study^86^, glucose infusion into the ileum, but not duodenum, suppresses food intake, and a rodent study similarly found that ileal glucose infusion suppresses food intake to a greater degree than duodenal glucose infusion^87^. These studies support the notion that ileal nutrient-sensing regulates gut motility^88^ but later proposed by many to also regulate food intake^89^. In contrast to the upper small intestine, this may be due to SGLT-1 independent glucose-mediated GLP-1 release^90^ (Fig. 2b).

#### Effect of carbohydrate-sensing on glucose homeostasis

Small intestine infusion of glucose impacts glucose homeostasis and the effects are not only due to glucose absorption into circulation. First, it is well established that the GI tract contributes to insulin secretion via the incretin effects of GLP-1 and GIP, which stimulate insulin secretion from the pancreas. The incretin effect may account for up to 80% of total insulin secreted in response to an oral glucose tolerance test^14^. Direct infusion of glucose into the duodenum in humans also increases circulating insulin levels, as does jejunal infusions, while glucagon levels either decrease or remain unchanged^91^. This discrepancy in glucagon is likely due to the differing actions of GIP and GLP-1, as GIP paradoxically increases while GLP-1 inhibits glucagon secretion^15^. While both GLP-1R and GIPR knockout mice exhibit reduced insulin release in response to intestinal glucose, each model only exhibits mild glucose intolerance. However, dual GLP-1R and GIPR knockout mice exhibit substantially impaired glycemic control and oral glucose-stimulated insulin release as compared to single incretin receptor knockout mice^92^. A similar additive result on glucose excursions is observed in humans treated with GIPR and/or GLP-1R antagonists^93^. Further, GIP was found to be a more powerful incretin hormone than GLP-1, but its overall effect on glucose homeostasis is likely masked by the concomitant rise in glucagon^93^.

Owing to the short half-life of GLP-1 (~1–2 min), only ~12% of the GLP-1 secreted from the gut enters the systemic circulation intact and activates the GLP-1R expressed in the pancreas^21^. As such, the common hepatic branch of the vagus, as well as celiac and gastric branches, are all implicated in contributing to the glucoregulatory effects of GLP-1 action^94,95^. For example, selective knockdown of GLP-1R in the nodose ganglia impairs glucose response to a mixed meal but interestingly does not impair oral glucose tolerance^22^. This implies that the impaired response to a mixed meal challenge is not dependent on altered intestinal glucose-sensing. Further, the impact of genetic knockout of GLP-1R in vagal neurons on oral glucose tolerance is contentious^22,23^. However, selective restoration of the islet and pancreatic duct GLP-1R in global GLP-1R knockout mice was sufficient to improve impaired oral glucose tolerance, although the reason for this is unknown as there was no change in glucose-stimulated insulin release among the groups^96^. Thus, the mechanism of glucose-induced GLP-1 regulation on insulin secretion remains elusive.

Direct infusion of glucose into the upper small intestine or jejunum given at a dose that does not increase portal glucose levels activates small intestinal SGLT-1 and lowers hepatic glucose production in parallel to an increase in portal GLP-1 levels^97,98^ (Fig. 2b). Similar to the mechanism of glucose-sensing in the regulation of food intake, infusion of non-metabolizable sugar 3-OMG (that is transported via SGLT-1) into the upper small intestine recapitulates the glucoregulatory effect of glucose-sensing^97^, suggesting that upper small intestinal glucose-sensing in inducing GLP-1 release is dependent on the electrogenic capacity of SGLT-1 but independent of cellular glucose metabolism. Further, the effect of small intestinal glucose sensing on hepatic glucose production regulation is abolished when glucose is co-infused with GLP-1R antagonist exendin-9^97^, strengthening the role of GLP-1 as the mediator of intestinal glucose-sensing on hepatic glucose production^99^.

#### Effect of metabolic dysregulation on small intestinal carbohydrate sensing

Despite the prevalence of carbohydrates in the diet, few studies have investigated the effect of obesity or HFD on intestinal glucose sensing. In rodents, both diet-induced and genetic models of obesity exhibit reduced satiation in response to intraduodenal carbohydrate infusion, although the effect is less pronounced than what is observed with intestinal lipids and is observed in some but not all studies^57,58^. Moreover, there are no differences in the response to duodenal infusion of glucose between participants with and without obesity^100^. Thus, it is likely that a high-fat diet and/or obesity do not greatly impair intestinal carbohydrate sensing and subsequent regulation of food intake. In contrast, obesity is associated with reduced postprandial GLP-1 levels^101^ and sensitivity to GLP-1 in rodents^102,103^, although gut peptides other than GLP-1 may mediate the anorectic effect of intestinal carbohydrates^104^. Despite these unknowns, research with human participants suggests that the incretin effect is impaired in diabetes, which is likely due to reduced GLP-1 secretion and impaired potency of GLP-1 to induce insulin secretion^105^. Similarly, HFD in rodents impairs the ability of upper small intestinal glucose infusion to lower glucose production, likely due to reduced GLP-1 secretion^97^. This reduction in GLP-1 secretion during HFD is associated with decreased upper small intestinal SGLT-1 levels^97^. In line with this, HFD reduces SGLT1 expression in small intestinal L-cells, resulting in impaired GLP-1 response to glucose in primary cultures^106^ (Fig. 2b).

### Small intestinal protein sensing

#### Effect of protein-sensing on food intake

High protein diets in both humans and rodents reduce body weight and adiposity in association with intestinal protein sensing-related increases in gut peptide levels. In humans, duodenal infusion of whey protein hydrolysate decreases food intake without a change in subjective appetite ratings^107,108^ but in parallel to increased GLP-1 and CCK levels^107,109^. In addition, casein infusion into the ileum of humans also decreases food intake, whereas infusion into the duodenum or jejunum has minimal effect. This is possibly explained by the fact that ileal casein infusion resulted in the greatest rise in GLP-1 levels compared to duodenal or jejunal infusion^110^. In rodents, various protein solutions potentially reduce food intake more potently than isocaloric and isovolumetric carbohydrate infusions^111^, and the underlying mechanisms may involve CCK release and subsequent activation of CCK-1R on vagal afferent neurons^112–114^, although GLP-1R signaling was not investigated. Thus, future studies are needed to more definitively identify the specific effects of different types of protein and the intestinal site of protein sensing on the regulation of food intake and gut peptide release.

#### Effect of protein-sensing on glucose homeostasis

High protein diets improve glucose homeostasis in both rodents and humans^115,116^, even in the absence of weight loss in patients with diabetes or during pair-feeding in rodents^117,118^. In humans, duodenal whey protein hydrolysate impacts circulating glucose, insulin, and glucagon^107,108^, while duodenal, jejunal, or ileal casein infusion leads to a substantial increase in insulin levels with no change in glucose^110^. Moreover, infusion of leucine alone into the duodenum dose-dependently increases insulin, with slight decreases in glucose, but no change in glucagon^119^. In rodents, upper small intestinal infusion of casein hydrolysate for 50 min at levels that do not increase circulating amino acids levels increases glucose tolerance and reduces glucose production during a pancreatic euglycemic clamp^120^. These effects are mediated by peptide transporter-1 (PepT1), a di- and tri-peptide proton-coupled transporter located in the brush border membrane of the intestinal epithelium^120^ (Fig. 2c). PepT1 mediates the secretion of GLP-1 from small intestinal EECs^121^ and CCK/GLP-1 secretion in vivo^122^, possibly via depolarization of EECs during transport of peptides into the cell in a similar fashion as the glucose-SGLT-1 axis^121,123^. Recent evidence using isolated intestinal perfusion technique indicates that dietary protein induces gut peptide secretion via transport of oligopeptides into cells via PepT1. Cellular oligopeptides are broken down into individual amino acids that are released to the basolateral side of the intestine to activate amino acid receptors^123^. In addition, amino acids induce gut peptide release, both in vivo and in vitro, via the calcium-sensing receptor (CaSR)^124^, the umami taste receptor (Tas1R1/Tas1R3)^125^, and G protein-coupled receptor family c group 6 member A^126^. Taken together, these data indicate that both apical PepT1 and basolateral CaSR could be critical for peptone-mediated GLP-1 release (Fig. 2c). Nonetheless, more work is needed to determine the exact mechanism linking intestinal protein sensing to gut peptide release, and which specific amino acids and sensors are required.

#### Effect of metabolic dysregulation on small intestinal protein-sensing

In contrast to lipids and carbohydrates, sensitivity to intestinal protein-sensing appears to be maintained during obesity, highlighting the potential of protein-sensing as a therapeutic target for weight loss. There are no differences in energy intake or CCK and GLP-1 responses between individuals with and without obesity following intraduodenal whey protein infusion^127^. In line with this data, rats fed an HFD for either 3 or 28 days, with the latter resulting in increased adiposity, still responded to small intestinal casein infusion by lowering hepatic glucose production^120^. In addition, high protein intake improves metabolic outcomes, like body weight, adiposity, insulin sensitivity, and food intake, in both rodents and humans^128–130^, and improves glucose tolerance and lowers blood glucose levels in patients with diabetes^131^. This may be explained by the fact that intestinal proteins more potently stimulate gut peptide secretion as compared to isocaloric lipids or carbohydrates^132^. Future research is warranted to uncover the mechanisms of how intestinal protein sensing, but not lipid or carbohydrate sensing, is maintained during metabolic dysregulation.

### Role of gut microbiota on small intestinal nutrient sensing and therapeutics

**Fig. 3:**
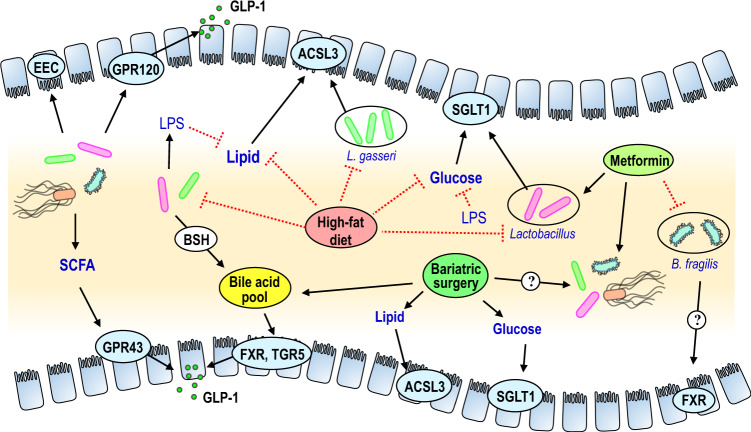
Interaction of gut microbiota and small intestinal nutrient sensing. We put forward a working hypothesis for the mechanistic links between small intestinal nutrient-sensing, microbiota, peptide release, and metabolic regulation. (From Left to Right of the Schematic): Bacterial species can directly and/or indirectly impact epithelial GPRs to alter GLP1 expression and release. Bacterial by-products such as LPS can impair lipid and glucose sensing and potentially disrupt ACSL3 and SGLT1 dependent pathways that regulate glucose and energy homeostasis. Bile salt hydrolase of bacteria contributes to the bile acid pool and regulates bile acid metabolism. As a result, changes in bile acids can alter GLP-1 release and metabolic regulation via intestinal FXR and TGR5 signaling. High-fat feeding reduces the abundance of small intestinal *Lactobacillus* species (e.g., *L. gasseri*) and consequently inhibits ACSL3 expression and impairs lipid sensing. Lastly, metformin increases the abundance of upper small intestinal *Lactobacillus* and enhances SGLT1 expression and glucose sensing, while also reducing the abundance of *Bacteroides fragilis* that results in ileal FXR inhibition and improvement in glucose metabolism. Bariatric surgery enhances small intestinal nutrient sensing mechanisms and consequently lowers glucose levels, while changes in bile acid metabolism and FXR are necessary for the glucose-lowering effect of bariatric surgery.

Changes in the gut microbiota affect obesity and related metabolic disorders, and the mechanisms linking the gut microbiota to energy and glucose homeostasis have been extensively reviewed^133,134^. However, the majority of the studies have focused on the role of the microbiota in the large intestine, and few studies have examined the metabolic impact of the small intestinal microbiota. While there are several orders of magnitude greater abundance of bacteria in the large intestine than in the small intestine, nutrient-sensing, and gut–brain feedback mechanisms are localized to the small intestine, as nutrient absorption limits ingested macronutrients from reaching the large intestine. Further, the protective barrier of a mucus layer in the small intestine is much less established^135^, allowing for an increased potential for intimate interactions between the host epithelial cells and the gut bacteria. For example, restoring the gut microbiome in germ-free mice results in an acute, transient phase, followed by a homeostatic phase that impacts jejunal transcriptomics and metabolomics involved in lipid and glucose metabolism and uptake^136^. However, the initial acute response is not observed in the ileum or colon, highlighting the sensitivity of the upper small intestine to the microbiome. Evidence suggests that the microbiota could also greatly impact nutrient-sensing mechanisms. First, microbial metabolites, especially short-chain fatty acids (SCFAs), are known to induce gut peptide secretion from EECs^137,138^. Most bacterially derived metabolites like SCFAs are produced predominantly in the distal intestine but are also present in small amounts in the ileum and can reduce glucose production via a gut–brain axis^139,140^. Other metabolites, like indole, are highly abundant in the small intestine and also regulate GLP-1 release from EECs^141^. Secondly, the gut microbiota impacts EEC physiology. For example, isolated cells expressing GLP-1 obtained from germ-free and conventional mice exhibit different transcriptomes, which is rapidly altered after only one day of microbiome colonization, suggesting a more direct effect of the bacteria on the EECs vs. an indirect effect from altered physiology of the germ-free model^142^. Further, intestinal expression and circulating levels of gut peptides are altered in germ-free mice^143,144^. Similarly, HFD converts zebrafish EECs into a nutrient-insensitive state dependent on gut microbiota, as germ-free zebrafish are resistant to the induction of EEC nutrient-insensitivity while an *Acinetobacter* strain was able to induce EEC nutrient-insensitivity^145^. In line with this, bacterial species directly influence GPR120, a receptor linked with lipid-induced gut peptide secretion, and GLP-1 expression in vitro^146^. Third, LPS, a bacterial byproduct, blunts vagal activation by intestinal nutrients, leptin, or CCK^147,148^. Thus, there exists a precedent for the ability of small intestinal microbiota to impact nutrient-induced small intestinal gut–brain signaling (Fig. 3).

In parallel, gut microbiota alters the bile acid pool and thereby potentially affects nutrient sensing and glucose and energy homeostasis. Conjugated bile acids are produced in the liver and released into the duodenum, where they are either absorbed or de-conjugated by the bile salt hydrolase of bacteria. Bile acids act as signaling molecules in the intestine and elsewhere, binding to FXR and G protein-coupled receptor 19 (also known as TGR5)^149^. Most, but not all, studies indicate that inhibition of intestinal FXR improves energy and glucose homeostasis^150^, and FXR signaling represses transcription of GLP-1 and inhibits GLP-1 release from L-cells^151^. Interestingly, TGR5 signaling increases GLP-1 release from L-cells^152^, thus complicating the role of bile acid signaling in the intestine (Fig. 3).

HF-feeding, obesity, and diabetes are all associated with unique microbial profiles in the large intestine. However, evidence suggests that HF-feeding also alters the composition of small intestinal gut microbiota. In rodents, the majority of the small intestinal bacteria are *Lactobacillius*, and HF-feeding results in a drastic reduction in the relative abundance of this genus^45,97^. Recent work indicates that altered small intestinal microbiota during HFD drives impairments in intestinal lipid-sensing, as the transplant of the small intestinal microbiota of short-term HF fed rats into chow-fed rats abolished the ability of small intestinal lipid infusion to improve glucose tolerance and lower hepatic glucose production. Treatment of HF-fed rats with a small intestinal infusion of *Lactobacillus gasseri* enhances upper small intestinal lipid-sensing, via restoration of long-chain acyl-CoA synthetase (ACSL3)^45^. *L. gasseri* exhibits bile salt hydrolase activity^153^ and can thus alter the composition of the bile acid pool. Small intestinal *L. gasseri* increases ACSL3 and subsequent lipid-sensing through a mechanism dependent on reduced FXR signaling.^45^. These findings are consistent with the fact that bile acid sequestrants (i.e., colesevelam) that inhibit FXR improve glycemic control^154^ (Fig. 3).

Recent evidence-based on studies with the anti-diabetic medicine metformin indicate that the glucoregulatory impact of intestinal glucose-sensing is mediated by the small intestinal microbiota. While metformin directly influences hepatic metabolism^155^, as an orally administered drug metformin concentrations in the small intestine are much greater than in the serum^156^. Oral metformin reduces blood glucose levels more than intravenous or portal vein administration^157^, demonstrating a role for intestinal-mediated mechanisms of action in improvements in glucose homeostasis. Pretreatment of HF-fed rats with metformin restores the ability of upper small intestinal glucose infusion to lower glucose production via increased portal vein GLP-1 levels and small intestinal SGLT-1 expression and in parallel changes the composition of small intestinal microbiota^97^. This is in line with several other studies that highlight the importance of the gut microbiota in mediating the beneficial effects of metformin^158,159^. In addition, individuals with newly diagnosed diabetes treated with metformin for three days exhibit alterations in the gut microbiota including increased *Lactobacillus* and reduced *Bacteroides fragilis* abundance, which result in inhibition of FXR signaling to improve glucose metabolism^158^. This observation is similar to the ability of *L. gasseri* to increase intestinal lipid-sensing to improve glucose homeostasis via FXR^45^(Fig. 3). Collectively, these studies highlight small intestinal nutrient-sensing mechanism mediates the beneficial effects of metformin through changes in gut microbiota and bile acids.

Evidence is emerging on the impact of the small intestinal microbiota also in the efficacy of gastric bypass. Despite extensive evidence of an overall role of the large intestinal microbiota in mediating the effects of bariatric surgery^160^, at least one study demonstrated that gastric bypass alters the microbiota of the duodenum, jejunum, and ileum^161^. In addition, while the jejunal nutrient-sensing mechanism at least partly mediates the beneficial effects of duodenal–jejunal bypass surgery on glucose homeostasis^98^, the glucose-lowering effect of vertical sleeve gastrectomy is dependent on both the gut microbiota and bile acid signaling^162^ (Fig. 3).

## New avenues for research and conclusions

While technological advancements begin to detail the role of intestinal nutrient-sensing in gut–brain neuronal signaling, they concurrently expand the field. One example of this is the use of single-cell RNA sequencing to understand vagal afferent signaling. Several groups distinctly labeled nodose ganglion neurons according to their expression profile, however, the results are expansive and sometimes contradictory^44,163^. Based on these studies, vagal afferent neurons containing GLP-1R have no impact on intestinal nutrient-sensing mechanisms, which are instead regulated by GPR65-positive neurons^44^. Despite being activated by intestinal nutrients, direct optogenetic or chemogenetic activation of GPR65 + nodose neuronal cells has little effect on food intake, thus it is still unknown which vagal neurons regulate the suppressive effects of intestinal nutrients^163^. Indeed, various neurons terminating in the intestinal mucosa, that likely sense gut peptides released in response to intestinal nutrients, have no effect on food intake, and only direct activation of a subset of IGLE neurons that detect intestinal stretch and not gut peptides suppresses food intake^163^. A subset of EECs called neuropods exist that directly synapse with vagal neurons, and rapidly signal via glutamate to the nucleus of the solitary tract in a single synapse to relay initial spatial and temporal information about the meal that could later be followed by more traditional gut peptide signaling^164^. Despite these interesting and exciting advances and the discovery of new nutrient sensory cells, the exact neurons that mediate the gut–brain signaling and nutrient sensing in regulating metabolism are complex and warrant future investigations. Future studies are needed to start teasing apart these complexities, while also integrating the gut microbiota and metabolites into the picture. For instance, while the gut microbiota can impact EECs, it is plausible that vagal afferents themselves can be impacted by bacterial metabolites^165^.

In contrast to energy intake, the impact of nutrient-induced gut–brain vagal signaling on energy expenditure has been poorly characterized. Intestinal lipids regulate brown fat thermogenesis via vagal afferents^166^ and possibly via GLP-1R signaling^167^, and vagal knockout of the transcription factor peroxisome proliferator-activated receptor-γ, which is activated by fatty acids and could thus be involved in lipid-sensing, affects thermogenesis^168^. Likewise in humans, intraduodenal infusion of intralipid increases resting energy expenditure^52^. Nutrient infusions into the duodenum of rats modulate energy expenditure^169^. Future work is needed to detail the connections between nutrient-sensing mechanism, gut microbiota, and impact on energy expenditure via thermogenesis in brown or browning white adipose tissue^170^.

Overall, extensive evidence indicates that targeting nutrient sensing in the small intestine impacts energy and glucose homeostasis during normal physiology and in the context of obesity and type 2 diabetes. Given the distinct effects of HFD and obesity on the diminution of nutrient-sensing dependent gut–brain pathways, future studies examining the gene and environmental interactions are warranted to further the development of personalized medicine approaches. Similarly, the expansive role of the gut microbiota in host metabolic health further highlights the need for personalized approaches to treating metabolic diseases. As such, studies in humans and rodents beginning to unravel the interactions between the gut microbiota, small intestinal EECs, and vagal signaling, are laying the groundwork for the development of therapeutics targeting small intestinal nutrient sensing to treat obesity and type 2 diabetes.
